# Classification of multi-year and multi-variety pumpkin seeds using hyperspectral imaging technology and three-dimensional convolutional neural network

**DOI:** 10.1186/s13007-023-01057-3

**Published:** 2023-08-10

**Authors:** Xiyao Li, Xuping Feng, Hui Fang, Ningyuan Yang, Guofeng Yang, Zeyu Yu, Jia Shen, Wei Geng, Yong He

**Affiliations:** 1 College of Biosystems Engineering and Food Science, Zhejiang University, Hangzhou, 310058 China; 2 The Rural Development Academy, Zhejiang University, Hangzhou, 310058 China; 3 Institute of Vegetables, Zhejiang Academy of Agricultural Sciences, Hangzhou, 310000 China

**Keywords:** Classification, Seed, Hyperspectral imaging, Deep learning

## Abstract

**Background:**

Pumpkin seeds are major oil crops with high nutritional value and high oil content. The collection and identification of different pumpkin germplasm resources play a significant role in the realization of precision breeding and variety improvement. In this research, we collected 75 species of pumpkin from the Zhejiang Province of China. 35,927 near-infrared hyperspectral images of 75 types of pumpkin seeds were used as the research object.

**Results:**

To realize the rapid classification of pumpkin seed varieties, position attention embedded three-dimensional convolutional neural network (PA-3DCNN) was designed based on hyperspectral image technology. The experimental results showed that PA-3DCNN had the best classification effect than other classical machine learning technology. The classification accuracy of 99.14% and 95.20% were severally reached on the training and test sets. We also demonstrated that the PA-3DCNN model performed well in next year’s classification with fine-tuning and met with 94.8% accuracy.

**Conclusions:**

The model performance improved by introducing double convolution and pooling structure and position attention module. Meanwhile, the generalization performance of the model was verified, which can be adopted for the classification of pumpkin seeds in multiple years. This study provided a new strategy and a feasible technical approach for identifying germplasm resources of pumpkin seeds.

## Background

Pumpkin is a cucurbit crop with a wide range of plantings and a wide variety of resources [1]. Excavating high-yielding pumpkin germplasm resources will greatly enrich the edible oil market, which is of practical significance to increase the added value of pumpkin. In addition, pumpkin seeds are rich in amino acids, proteins and trace elements, which have high nutritional value and momentous research value [2–5]. However, due to the late start of breeding research and the narrow genetic background of germplasm resources, the breeding efficiency of seed-used pumpkins whose seeds as the main edible organs or processing objects needs to be improved urgently. The classification of pumpkin seed varieties is an important link in the identification of germplasm resources, which can effectively improve the purity of varieties, increase crop yield and promote breeding improvement [6, 7]. At the same time, it is of great significance to further realize the rapid screening of high-quality trait genes.

Traditional manual inspection and machine vision techniques for variety classification and grading are time-consuming and labor-intensive. It no longer meets the current requirements of high efficiency, accuracy and no damage [8, 9]. Hyperspectral imaging (HSI) technology can get rich spectral data and spatial information of seed spectral images at the same time, which is widely employed in the seed classification field [10–13]. In particular, compared with the visible light bands, the mid-infrared bands, etc., the near-infrared (NIR) spectral region is consistent with the absorption region of the combination frequency and double frequency of the vibration of hydrogen-containing groups (O**–**H, N**–**H, C**–**H) in organic molecules [14]. By obtaining the NIR spectrum of pumpkin seeds, the characteristic information of hydrogen-containing groups can be acquired, so as to precisely reflect the composition and properties of the seeds for accurate classification. Traditional methods usually took advantage of principal component analysis and other methods to select or transform original spectral features, and then adopted traditional machine learning methods such as support vector machine (SVM) to train classifier models [15–19]. The separation of feature learning and classifiers easily led to the ineffective extraction of features. The compressed features thereby may not guarantee the classification accuracy. Additionally, traditional machine learning generally assumes that the samples for training and testing have no relation and have the same distribution, which is why the model obtained on the training set can be equally effective on the test set. As a consequence, in the case of different domains and tasks, such as the classification of multi-year seeds, traditional machine learning is limited.

As a new study direction in machine learning, deep learning can automatically learn features through computers and has excellent feature extraction capabilities [20–26]. Convolutional Neural Network (CNN) is one of the typical and commonly used models and has been gradually applied to spectral analysis recently [27–29]. At present, CNN constructed based on HSI can be roughly divided into one-dimensional convolutional neural networks (1DCNN), two-dimensional convolutional neural networks (2DCNN) and three-dimensional convolutional neural networks (3DCNN) [30]. 1DCNN is constructed from one-dimensional (1D) averaged spectra because spectral information is the most important feature of HIS [31, 32]. Although such models had achieved good results, the classification accuracy still depended on the adequacy of manually extracted features. Moreover, keeping only average spectra may also result in suboptimal model performance. 2DCNN is specially designed for RGB images, so it pays attention to extracting spatial features from raw spectral images [33]. Although the seed classification performance of 2DCNN was better than that of 1DCNN in some studies [34]. But extracting and compressing classification features using two-dimensional (2D) convolutions hardly took into account the spatial and spectral dimensions of HSI data, which made it difficult for deep learning models to fully mine features required for classification.

HSI is presented in the form of three-dimensional (3D) data cubes that exhibit correlations in both spatial and spectral dimensions. It is troublesome for 2D convolution to completely exploit the feature coupling relationship between different bands in 3D HSI data. 3D convolution was first applied for human action recognition, which can extract features in both spatial and temporal dimensions. Compared with 2D convolution, it had better recognition and classification effect in the vast majority of cases [35–37]. Therefore, 3DCNN can straightly gain integrated deep spectral and spatial information from raw HSI in an end-to-end manner, which would validly ameliorate model accuracy [38–40]. Jung et al. designed 2DCNN and 3DCNN to identify susceptible areas, asymptomatic areas and healthy areas of strawberry leaves [41]. The classification accuracy of the latter was 84%, which was 10% higher than that of the former. Gao et al. used SVM and 3DCNN to classify heat-shocked rice seeds and normal rice seeds, and 3DCNN received a higher accuracy of 97.5% [42]. As a consequence, 3DCNN has enormous latent capacity in the identification and classification of crop seed varieties based on HSI.

From another perspective, deep networks need enough samples for each type of seed to fully learn to extract features hidden in redundant spectral data. When the amount of data required to build models is not available, the model may overfit or fall into a local optimum. In addition, due to the influence of various external environmental factors, spectral characteristics of seeds vary widely from year to year. Whether the model has the same excellent classification effect for multi-year seeds is a major indicator to test its generalization ability. Transfer learning, such as fine-tuning, provides an efficient solution [43, 44]. Zhu et al. fine-tuned pretrained models including AlexNet, ResNet18, Xception, InceptionV3, DenseNet201 and NASNetLarge to categorize 10 types of soybean seeds [45]. Among them, NASNetLarge reached up to the best classification accuracy of 97.2%. Wu et al. designed a deep learning model called VGG-MODEL and transferred it to 4 crop seeds for classification, all of which attained higher performance than traditional methods [46]. To sum up, transfer learning can be successfully utilized in the fast and accurate classification of crop seeds that are multi-year, multi-variety and sample-scarce.

Under such context, this paper aimed to develop a method for classifying pumpkin seeds based on NIR hyperspectral technology for automated and intelligent germplasm identification. We first verified the feasibility of classification through regression analysis of chemical components and spectral clustering analysis. Additional objectives were: (1) to construct a superior model called position attention embedded 3D convolutional neural network (PA-3DCNN) to classify 75 classes of pumpkin seeds; (2) to build a PA-3DCNN transfer model to classify pumpkin seeds in the second year to explore its transferability and generalization ability.

## Methods and materials

**Fig. 1:**
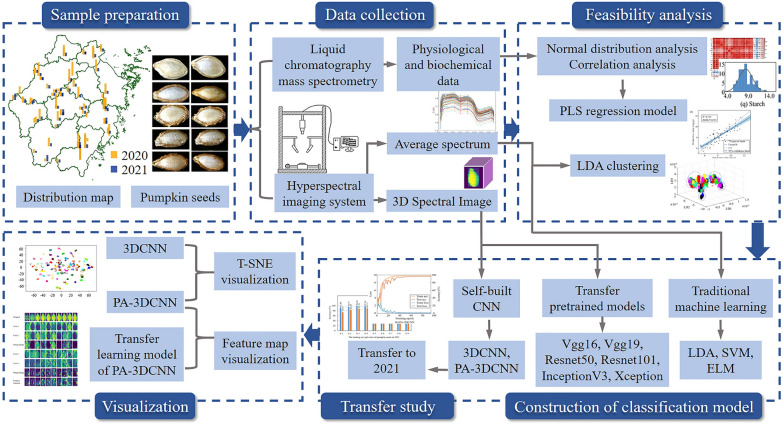
Flow chart of data analysis in this study

The main research process of this study included six parts: sample preparation, data collection, feasibility analysis, construction of classification model, transfer study and visualization (Fig. 1). Above all, the feasibility of classification was verified by collecting the chemical composition data and NIR spectral data of pumpkin seeds for two years. Subsequently, deep learning methods were exploited to build the pumpkin seed classification model. And the transferability of our model was verified by fine-tuning. Eventually, visualization was applied to explore the essence of spectral image classification.

### Sample preparation

The pumpkin seeds of 75 varieties belonging to different geographical locations in Zhejiang Province of China were collected by the Zhejiang Academy of Agricultural Sciences during 2017–2022. After the pumpkins in 2020 and 2021 were planted, their 75 and 56 types of seeds were separately collected as samples for this experiment.

All samples were uniformly packaged in kraft paper bags and stored in a dry room at a constant temperature of 23 °C. During the experiment, pumpkin seed samples with obvious defects such as damage, shriveled, and insect pests were excluded. And this study was conducted on the whole seed, including the shell.

### Physiological and biochemical data collection

The contents of physiological and biochemical components in different varieties of pumpkin seeds are different, which stimulates the diverse spectral fingerprints. Analyzing the degree of correlation between chemical composition content and spectral features of seeds is the premise of classification. The important components of pumpkin seeds are starch, soluble sugar, fat and protein. In addition, they contain rich amino acids, which are essential nutrients needed by the human body. Therefore, the chemical composition content including starch, fat, soluble sugar, total protein and 16 amino acid components (Asparagine, Threonine, Serine, Glycine, Alanine, Valine, Methionine, Isoleucine, Leucine, Tyrosine, Phenylalanine, Lysine, Histidine, Glutamine, Arginine, Proline) of 75 pumpkin seeds in 2020 were analyzed by liquid chromatography mass spectrometry (LC-MS1000; Jiangsu Skyray Instrument Co., Ltd., Kunshan City, China).

### Near-infrared hyperspectral imaging system

In this research process, the hyperspectral information of pumpkin seeds was acquired by near-infrared hyperspectral imaging system from 870 to 1740 nm. The system mainly consists of an imaging spectrometer (ImSpectorN17E; Spectral Imaging Ltd., Oulu, Finland), and a camera lens (OLES22; Specim, Spectral Imaging Ltd., Oulu, Finland). The specific structure is shown in Fig. 1. Other devices were introduced comprehensively in Feng et al. [47]. Before the formal experiment, the equipment was preheated for 30 min and the experimental parameters were adjusted and corrected. The exposure time was 3 ms. The distance between the lens and experimental sample was 19 cm. The speed of the displacement platform was 15 mm/s. The light intensity knob of the halogen lamp was adjusted to a stable state to ensure the integrity and clarity of the image information. After the acquisition, original spectral images were corrected in the HSI-Analyzer software using corrected images of black and white plates and the correction calculation formula described by Nie et al. [48].

In 2020, the valid samples number of 75 kinds of pumpkin seeds was 35,927. In 2021, that of 56 kinds of pumpkin seeds was 12,111. There was little difference in the effective sample size of different pumpkin varieties. The pumpkin seeds in 2020 were divided into training set and test set (7:3) at random in order to build classification models with excellent generalization ability. Figure 1 shows the visual distribution of geographic locations of various seeds in 2020 and 2021. The height of bars in the graph was proportional to the number of samples.

### Spectral data acquisition and preprocessing

Spectral data is provided by the near-infrared hyperspectral equipment in the form of a data cube. Therefore, the data can be analyzed in two different patterns, 1D spectral data and 3D spectral images. The pixels of all seeds were defined as regions of interest (ROI) and used as extraction targets to reduce the interference of background signals. The average spectrum of each pumpkin seed ROI was 1D spectral data. The smallest rectangular area used to extract and segment each seed ROI was 3D spectral images. The size of 3D spectral images of each seed was unified to 100 $\times$ 80 $\times$ 256 (length, width and number of bands of the image respectively) by symmetric zero-padding on both sides.

### Feasibility analysis methods for pumpkin seed classification based on NIR spectral characteristics

In order to explore the corresponding relationship between the spectral data and chemical components, the partial least squares (PLS) regression model was constructed to demonstrate feasibility of using NIR spectral information to identify pumpkin seeds. Correlation analysis and normal distribution analysis were carried out on 20 kinds of physiological and biochemical data of 75 kinds of pumpkin seeds. Normality was checked using the Kolmogorov**–**Smirnov test. The average spectrum was treated as independent variable in that regression model. The chemical components with a large proportion, strictly obeying the normal distribution and no obvious correlation were selected as the dependent variables.

At the same time, in order to further evaluate whether the spectral information was identifiable for pumpkin varieties, linear discriminant analysis (LDA) was employed to reduce the dimension and cluster the average spectral data of 75 pumpkin varieties. The above methods laid the foundation for building seed classification models.

### Construction of classification models based on average spectrum and traditional machine learning methods

In order to comply with the qualitative classification of 75 kinds of pumpkin seeds based on the average spectrum, LDA, SVM and extreme learning machine (ELM) machine learning methods were used to construct classification models respectively. LDA achieves the maximum distinguishability of samples in another space by calculating the correct projection direction and establishing a suitable linear discriminant function.

SVM distinguishes samples by building a nonlinear classification model and increasing the distance from the support vector to the hyperplane. In this study, the extremely stable radial basis function was selected. Penalty coefficient *c* and kernel parameter *g* were determined to be 1024 and 0.5 by using the toolbox function libsvm inside Matlab R2016a and grid search optimization.

ELM is a feedback-forward algorithm based on neural network. It contains three layers. The neurons number in the input, output layer was determined by the input and output of the original data itself. The number of hidden nodes was changed from 1 to 3000 in steps of 100, and the discriminative accuracy was computed. The final hidden nodes number *n* was determined to be 100.

### Construction of pumpkin seed classification models based on pretrained models

Using transfer learning to fine-tune pretrained models is faster and easier than randomly training weights from scratch. And it doesn’t require plenty of images. Accordingly, this study attempted to transfer six pretrained models including Vgg16, Vgg19, ResNet50, ResNet101, InceptionV3, and Xception to build classification models based on pumpkin seed spectral images. They are all classic two-dimensional convolutional neural networks formed by the continuous development of image classification in recent years. The depth and width of the networks are different, but they all have strong robustness and generalization performance.

To match three channels of RGB images, this study made use of principal component analysis to extract main components from 256 bands of original spectral images and retained the first three principal components. The target domain of this study was the HSI of pumpkin seeds, which was quite different from the source domain ImageNet dataset. Therefore, the weights in the first *k* layers of the pretrained model were respectively frozen, and the fully connected layer and output layer were adjusted for retraining. The above models all added two fully connected layers. The first layer consisted of 2048 neurons, and the second layer consisted of 1024 neurons. In order to alleviate the overfitting problem, L2 regularization and dropout layers were applied after the fully connected layers. The dropout ratio was 0.5, and L2 regularization was 0.001. The output layer was processed by the softmax multi-classification function. In addition, the six classification models all employed the multi-class cross-entropy loss function and stochastic gradient descent optimization algorithm to fairly compare model performance. The learning rate and momentum decay coefficients were 0.0001 and 0.94, respectively. The number of samples fed into the network at a time was 32. The number of iterations epoch was 100. The number of frozen layers *k* was determined according to multiple trainings, so that the model did not overfit and the classification accuracy was optimal. The specific parameters are listed in Table 1.

**Table 1:** The parameters of pretrained models to build pumpkin seed classification models

Transfer model	Vgg16	Vgg19	ResNet50	ResNet101	InceptionV3	Xception
Input image size	224 $\times$ 224 $\times$ 3					299 $\times$ 299 $\times$ 3
Transfer model layers *n*	16	19	50	101	46	36
Freeze Transfer model layers *k*	11	15	40	91	44	36

### Construction of pumpkin seed classification model PA-3DCNN based on double convolution and pooling structure and position attention module

**Fig. 2:**
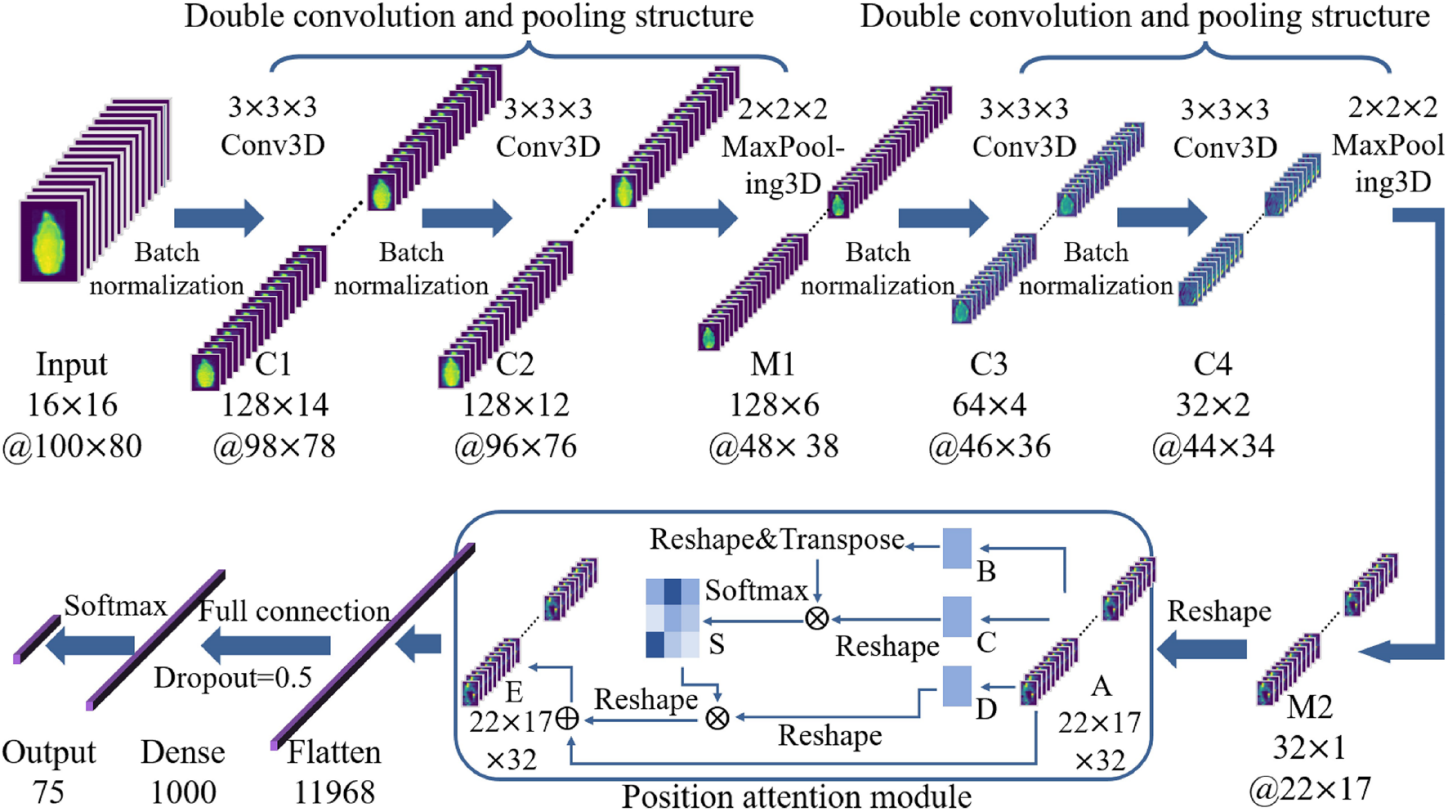
Model structure diagram of PA-3DCNN. The model consisted of 2 double convolution and pooling structures, the position attention module, fully connected layers, etc. Conv3D referred to the three-dimensional convolution operation. C1–C4 referred to the first to fourth convolutional layers. M1–M2 referred to the first and second pooling layers. A–E, S were the code names of the feature maps in the position attention module. Before @ was the number of feature maps, after which was the size of the feature map. Specific details were described in the paper

In contrast to 1DCNN or 2DCNN, 3DCNN directly processes the spectral data cube, thereby simultaneously processing spatial and spectral dimensions of pumpkin seed images. A three-dimensional convolution-based pumpkin seed classification model PA-3DCNN was constructed (Fig. 2). The input to the neural network was 16 spectral images of pumpkin seeds with a spatial dimension of 100 $\times$ 80 and a channel of 16.

Under the condition of network depth increased, the model classification accuracy decreases with the addition of convolutional layers. But limiting the model depth may reduce classification accuracy. Therefore, this study adopted 2 double convolution and pooling structures, which consisted of two convolutional layers, two batch normalization layers and one maxpooling layer. The pooling layers (M1, M2) were set after the double convolutional layer, and the batch normalization layer was set after each convolution for data normalization. Maxpooling3D pooling with a size of 2 $\times$ 2 $\times$ 2 was adopted for downsampling, which can effectively reduce the number of parameters and be more conducive to model convergence while maintaining feature invariance. However, frequent use would cause certain damage to spatial information. Hence, the double convolution design realized the maintenance and transmission of features. Moreover, experiments revealed that the 3 $\times$ 3 $\times$ 3 convolution kernel was a valid 3D convolution kernel for processing 3D spatiotemporal features [49]. So, 3 $\times$ 3 $\times$ 3 convolution kernels were utilized in this model. And the number of convolution kernels was continuously reduced as the depth of the neural network increased. According to experience and experiments, the first 2 convolutional layers (C1, C2) both used 128 channels, the third convolutional layer (C3) used 64 channels, and the fourth convolutional layer (C4) used 32 channels.

After four times of 3D convolution and two times of pooling, the spectral dimension was compressed to 1, and 3D convolution cannot be performed again. At this time, the size of feature map A is 22 $\times$ 17 $\times$ 32. Inspired by Fu et al. this study drew on the position attention module in the dual attention networks (DANet) [50]. By weighting all spatial features and selectively aggregating spatial features, the spatial interdependence of features was learned and the classification accuracy was improved. As shown in Fig. 2, feature map A was convolved three times to acquire three feature maps B, C, and D. Multiply the transpose of B by C, and then obtain the spatial attention map S through softmax. Then perform matrix multiplication on the transpose of D and S, and finally add to A to obtain the final feature map E. The output was flattened and input into a fully connected layer with 100 neurons. The dropout layer was applied to avoid overfitting. In this study, the dropout ratio was 0.5, and the output layer was processed by the softmax multi-classification function. The weights of convolution kernels were initialized by Xavier. And the multi-class cross-entropy loss function and stochastic gradient descent optimization algorithm were utilized. To verify the superiority of the model’s classification performance, PA-3DCNN and 3DCNN without position attention module were respectively trained and compared. Other parameters such as learning rate were the same as pretrained models.

### Transfer of pumpkin seed classification model PA-3DCNN

Due to the influence of various external environmental factors, the spectral characteristics of pumpkin seeds in 2021 and 2020 are quite dissimilar. The classification model constructed from the previous year’s images directly recognizes the seeds of the second year, which will inevitably lead to a significant drop in classification accuracy. In this study, the fine-tuning method of transfer learning was adopted, and spectral images of pumpkin seeds in 2021 were fed into PA-3DCNN at different scales. It allowed the model to re-adapt to new spectral features through low samples, thereby verifying the model’s generalization ability and realizing efficient classification of pumpkin seeds. All weights before the first double convolution and pooling structure of PA-3DCNN were frozen. Two fully connected layers and the output layer were retrained. The first and second fully connected layer’s neurons number was set to 1024 and 512. The output layer output 56 categories. In order to alleviate the overfitting problem, the dropout layer was applied after the fully connected layer, and the dropout ratio was 0.5. The number of samples input to the network at a time was 8. The settings of other parameters were the same as PA-3DCNN.

### Data visualization

Feature visualization can intuitively explore the feature extraction mode of CNN. The feature maps of the convolutional and pooling layers of PA-3DCNN were visualized as a single graph in each channel to explore the main points that the model concentrated on. In addition, the feature maps of the PA-3DCNN transfer model with a scale of 0.5 in the pumpkin seed training set in 2021 were in comparison with the original model feature maps to investigate the interpretability of feature extraction.

In order to visualize the classification process of 3D spectral images by deep learning, t-SNE was used to perform nonlinear dimensionality reduction on the feature data of the flattening layer, and subsequent layers. The t-SNE visualization of PA-3DCNN was compared with 3DCNN to explore the interpretability of the classification performance improvement by position attention mechanism.

### Software tools

Data analysis and model building were performed using a laboratory computer with Win10 64-bit operating system, Intel(R) Xeno(R) Gold 6242 CPU, 2.80 GHz, 128 GB RAM and Tesla V100. The extraction of the averaged spectrum and the construction of traditional machine learning models were performed in MATLAB R2016a. The deep learning framework Keras was adopted for the construction and training of convolutional network models. Figures were drawn based on OriginPro 9.0 (OriginLab Corporation, Northampton, MA, USA).

## Results and discussions

### Near-infrared spectroscopy of pumpkin seeds

**Fig. 3:**
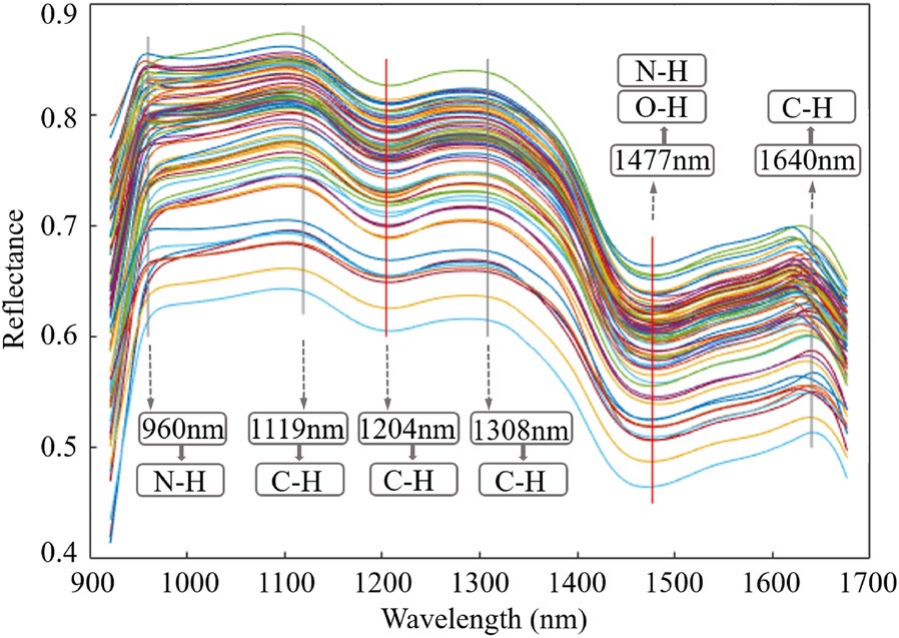
Average near-infrared spectral curves of 75 kinds of pumpkin seeds

The average NIR spectra of 75 types of pumpkin seeds ranging from 921.34 to 1676.33 nm are shown in Fig. 3. On the overall trend, these NIR spectral curves behaved consistently, but different species of seeds have distinct spectral reflectance due to genetic differences. The NIR spectral reflection is mainly generated by the vibration and rotation in the compound, including the biological macromolecular compound except for the fat of saturated higher fatty acid glyceride. Correspondingly, total protein and fat content in pumpkin seeds accounted for 60–70%. Soluble sugar and amino acid contented 20–30%. Starch content was less than 10%. Therefore, the mechanism of NIR action of pumpkin seeds and the absorption bands of spectral fingerprints were also very complicated. The molecular activity of specific spectral fingerprints was denoted on the straight line with marked concavities and convexities in Fig. 3. The peak around 960 nm was associated with the second-order frequency doubling of N–H bond stretching [51]. The peak at 1119 nm and the trough at 1204 nm had relation to that of C–H bond stretching [52]. The peak at 1308 nm was related to the combined frequency of C–H bond vibrations [53]. The trough at 1477 nm was related to the first-order frequency doubling of O–H bond and N–H bond stretching autocorrelation [54, 55]. The peak at 1640 nm had relation to the first-order frequency doubling of C–H bond [56]. It was the above-mentioned specific spectral features that made it possible to classify pumpkin seeds by NIR spectroscopy. However, due to the similar chemical composition of seeds and the lack of fundamental specific molecular differences, it was impossible to find specific spectral fingerprints of 75 kinds of pumpkin seeds from the single spectral curve. For this reason, further quantitative research was needed to achieve the classification and identification of seeds.

### Feasibility analysis results

**Fig. 4:**
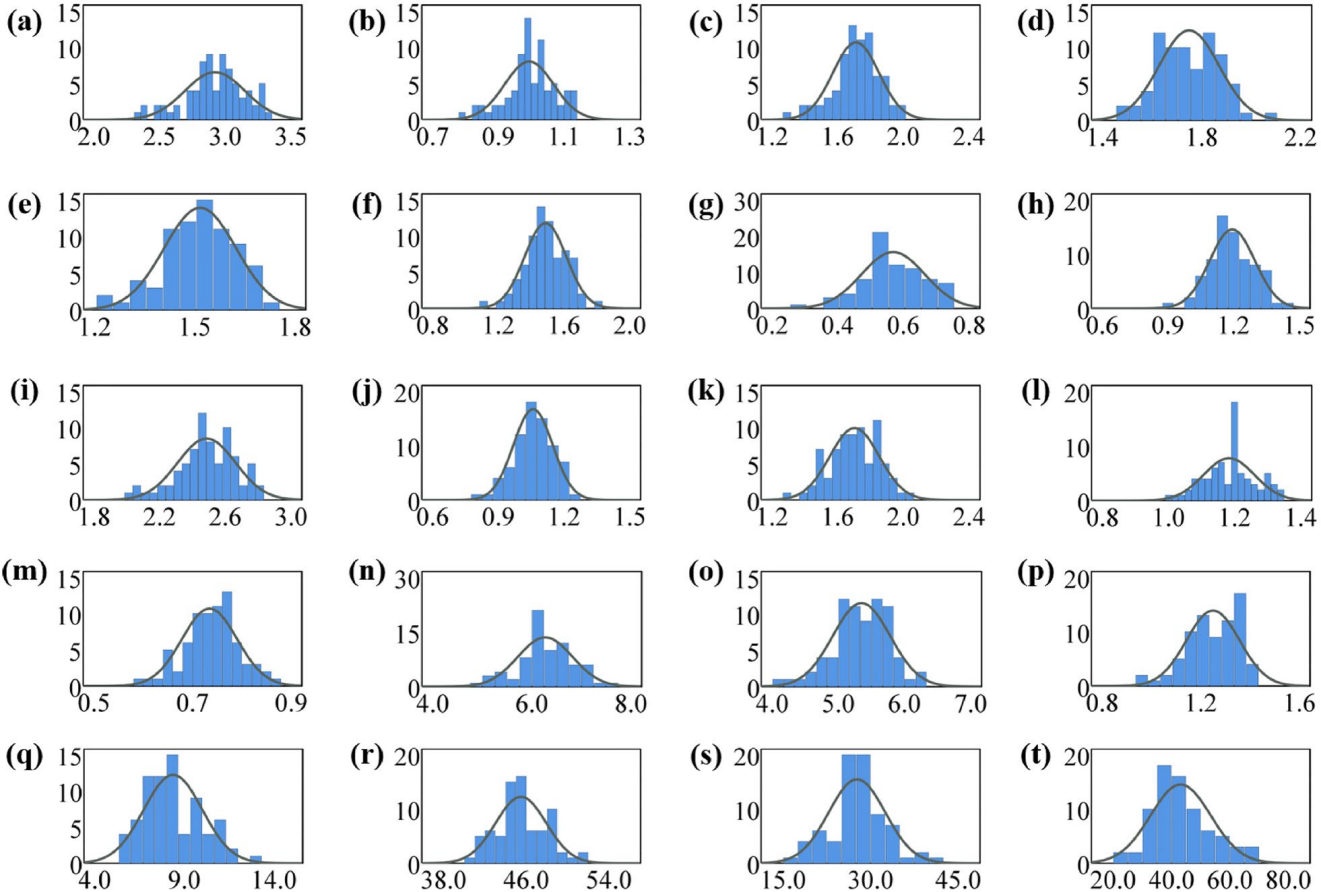
Normal distribution of 20 physiological and biochemical components of pumpkin seeds. **a** Asparagine, **b** Threonine, **c** Serine, **d** Glycine, **e** Alanine, **f** Valine, **g** Methionine, **h** Isoleucine, **i** Leucine, **j** Tyrosine, **k** Phenylalanine, **l** Lysine, **m** Histidine, **n** Glutamine, **o** Arginine, **p** Proline, **q** Starch, **r** Fat, **s** Soluble sugar, **t** Total protein

Descriptive statistical analysis was performed on the physiological and biochemical information of 75 kinds of pumpkin seeds. The normal distribution diagram is shown in Fig. 4 and data results are shown in Table 2. According to statistical results, the content of fat and total protein was the highest. Soluble sugar and starch accounted for a certain proportion. Amino acids occupied a small amount. The skewness coefficient of Asparagine was greater than 1.96, indicating that it did not obey the normal distribution. The significance level P of Threonine, Methionine and total protein was less than 0.05, which also believed that they did not obey the normal distribution. Correspondingly, other components obeyed the normal distribution.

**Table 2:** Statistical table and Kolmogorov–Smirnov test of physiological and biochemical components of pumpkin seeds

Physiological and biochemical components	Mean (mg/g)	Standard Deviation (mg/g)	Skewness coefficient	Kurtosis coefficient	Significance level P
Asparagine	2.920	0.229	1.964	0.303	0.200
Threonine	0.994	0.075	1.713	0.454	0.007**
Serine	1.711	0.141	1.881	0.615	0.200
Glycine	1.745	0.121	0.325	0.438	0.200
Alanine	1.516	0.108	1.668	0.144	0.200
Valine	1.487	0.127	0.538	0.489	0.200
Methionine	0.569	0.096	0.982	0.175	0.027*
Isoleucine	1.194	0.103	0.101	0.232	0.200
Leucine	2.486	0.177	1.917	0.385	0.200
Tyrosine	1.059	0.090	1.188	0.071	0.200
Phenylalanine	1.702	0.151	0.747	0.254	0.200
Lysine	1.183	0.077	0.170	0.562	0.085
Histidine	0.735	0.056	0.830	0.078	0.200
Glutamine	6.271	0.553	0.787	0.547	0.200
Arginine	5.349	0.435	1.621	0.666	0.200
Proline	1.250	0.108	1.866	0.248	0.190
Starch	8.365	1.635	1.726	0.297	0.067
Fat	45.474	2.447	1.134	0.219	0.191
Soluble sugar	27.669	4.843	0.036	0.721	0.185
Total protein	43.120	10.338	1.906	0.166	0.018*

*P refers to significant at the 0.05 level. **P refers to significant at the 0.01 level

**Fig. 5:**
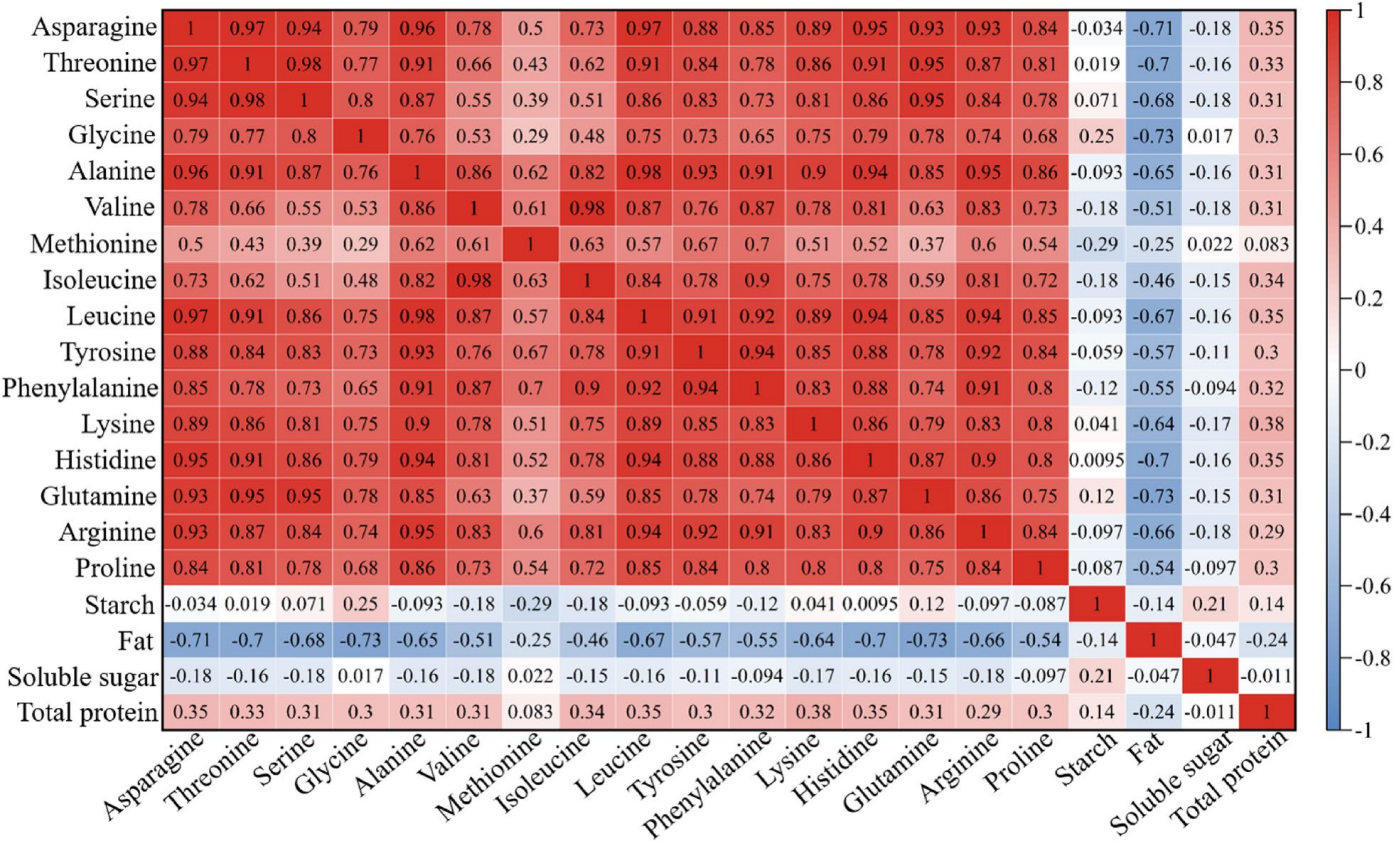
Correlation heat map of 20 physiological and biochemical components of pumpkin seeds

Correlation analysis was carried out on the physiological and biochemical information of 75 kinds of pumpkin seeds (Fig. 5). Among them, the highest correlation coefficient between starch, fat, soluble sugar and total protein was less than 0.3. The correlation coefficients of starch and soluble sugar with each amino acid were less than 0.2 or less, and there was no significant correlation. The overall correlation coefficient among the 16 amino acids was as high as 0.9, indicating a strong linear correlation. Locally, the average correlation coefficient between glycine and other amino acids was about 0.66, which was relatively low. In summary, four physiological and biochemical components with large proportions, strictly obeying normal distribution and no obvious correlation were selected for the establishment of PLS regression model. These four components were Glycine, starch, fat, and soluble sugar.

**Fig. 6:**
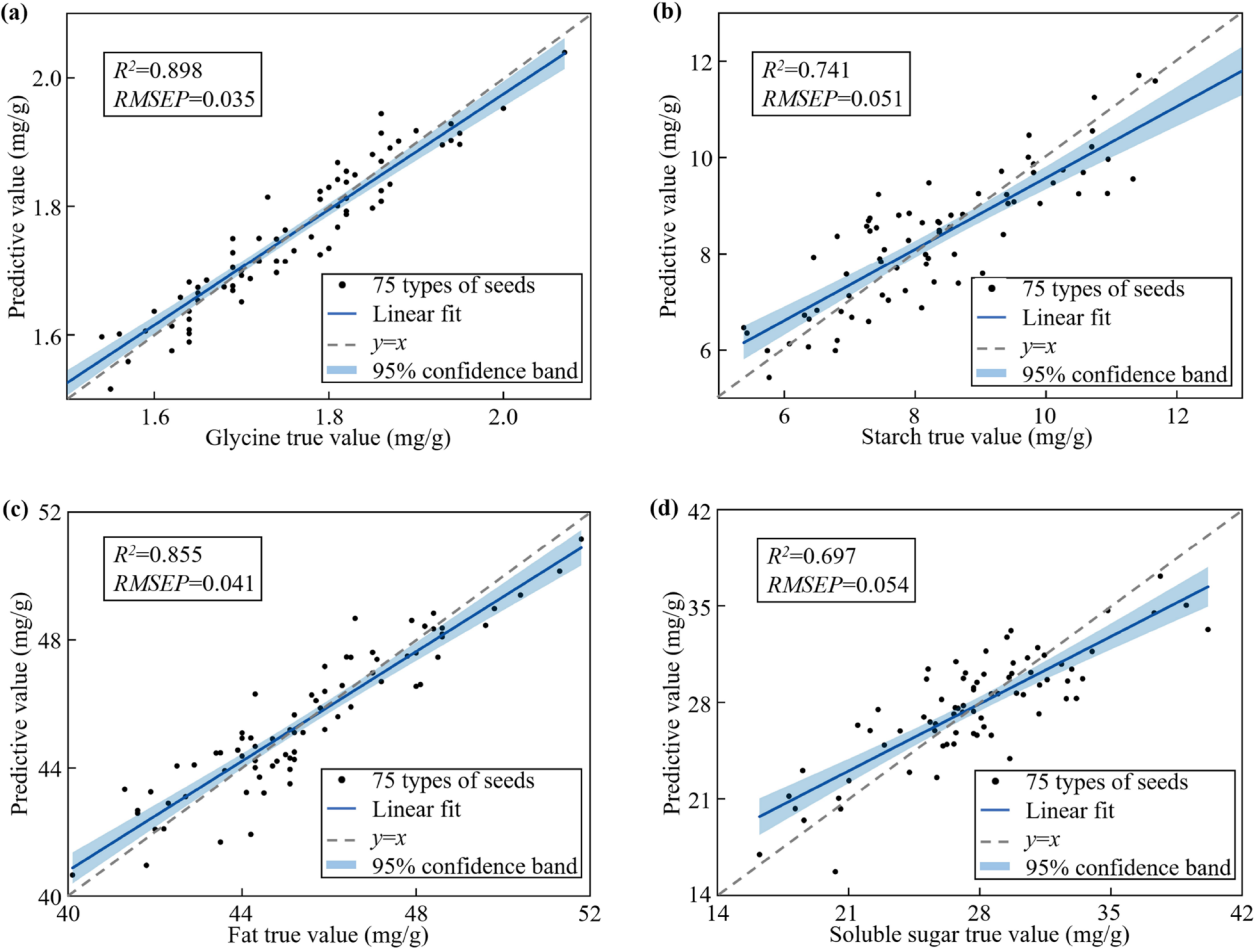
PLS regression model results for predicting four independent physiological and biochemical components based on near-infrared spectroscopy. **a** Correlation scatter plot of Glycine, **b** correlation scatter plot of starch,** c** correlation scatter plot of fat, **d **correlation scatter plot of soluble sugar

The PLS regression model was constructed for four chemical components on the average NIR spectrum of pumpkin seeds in the 921.34–1676.33 nm band. Figure 6 showed the scatter plot of the correlation between model predicted value and true value of the four components. On the whole, the coefficient of determination *R*^*2*^ of the regression model was above 0.65. Among them, the straight line fitting in Fig. 6a had the best effect. *R*^*2*^ and *RMSEP* also represented the strongest correlation of glycine content with the smallest error. In contrast, soluble sugar had a lower correlation of 0.697 with a maximum error of 0.054. In general, the regression model for four components all measured up satisfactory prediction results, which verified the strong correspondence between spectral fingerprint characteristics and chemical components of pumpkin seeds. Consequently, it was feasible to take advantage of NIR spectral data to identify pumpkin seeds.

**Fig. 7:**
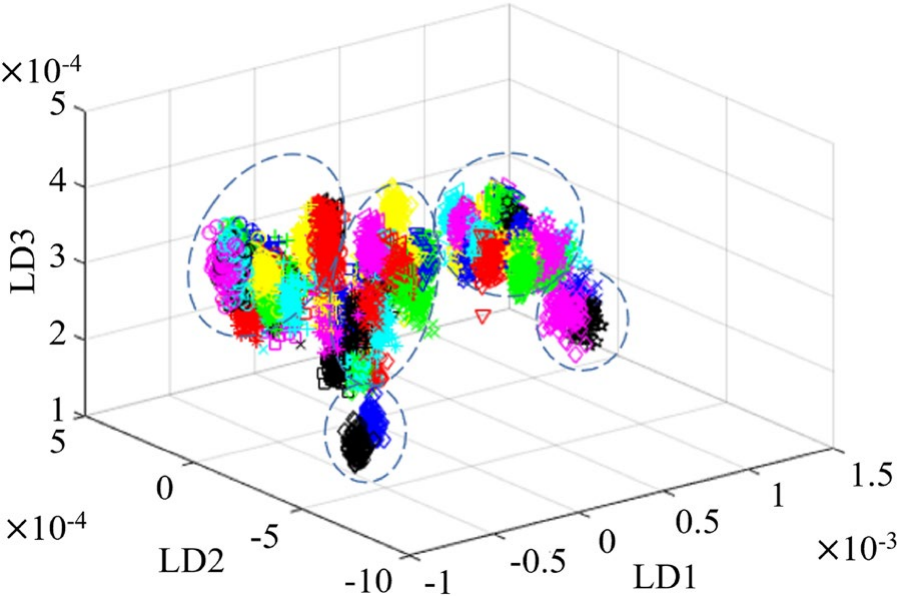
Visual clustering diagram of pumpkin seeds varieties based on LDA. Different varieties were identified according to the symbols and colors of 12 shapes and 7 colors

In addition, LDA was performed on the NIR average spectral data of 75 types of pumpkin seeds (Fig. 7). It was clear that LDA had obtained excellent clustering results in the dimensionality reduction of the NIR spectrum, which further verified the effectiveness and possibility of NIR spectral information for distinguishing seed varieties, and supplied a theoretical basis for building classification models. The samples of each variety were relatively concentrated and the boundaries between different kinds of pumpkin seeds were also relatively clear. On the whole, 75 kinds of pumpkin seeds were clustered in five groups, but accurate multi-variety classification cannot be achieved. The reason was that the classification accuracy of traditional methods for large sample datasets would decrease due to the simplicity of the algorithm itself. Deep learning is currently one of the best methods for big data processing and analysis. Therefore, the method would be used to classify pumpkin seeds in the following text.

### Results of pumpkin seed classification models based on average spectrum and traditional machine learning methods

LDA, SVM and ELM were constructed based on average spectral information of the full spectrum band of pumpkin seeds extracted from near-infrared hyperspectral images. The classification results are listed in Table 3. Three models have met with good results in the accuracy and error. ELM classification result was the best and reached 93.22%, and it was the most stable with an error of only 0.28. LDA and SVM also obtained test accuracies as high as 89.4% and 91.6%. It efficiently embodied the enhanced interaction between chemical components of the seeds and NIR spectrum. Optical electromagnetic waves in the NIR range not only cause electron transitions in molecules, but also vibrational and rotational transitions, such as the combined frequency and frequency doubled vibrations of organic compounds containing hydrogen groups. NIR spectral information can truly and validly reflect changes in chemical bonds, and then reach up to the purpose of accurately classifying pumpkin seeds.

**Table 3:** Classification results of LDA, SVM and ELM models based on near-infrared average spectra

Model	Training set accuracy (%)	Test set accuracy (%)	Time (s)	Error
LDA	91.60	89.40	232.6	0.39
SVM	92.44	91.60	516.6	0.33
ELM	96.40	93.22	102.6	0.28

### Results of pumpkin seed classification models based on pretrained models

**Fig. 8:**
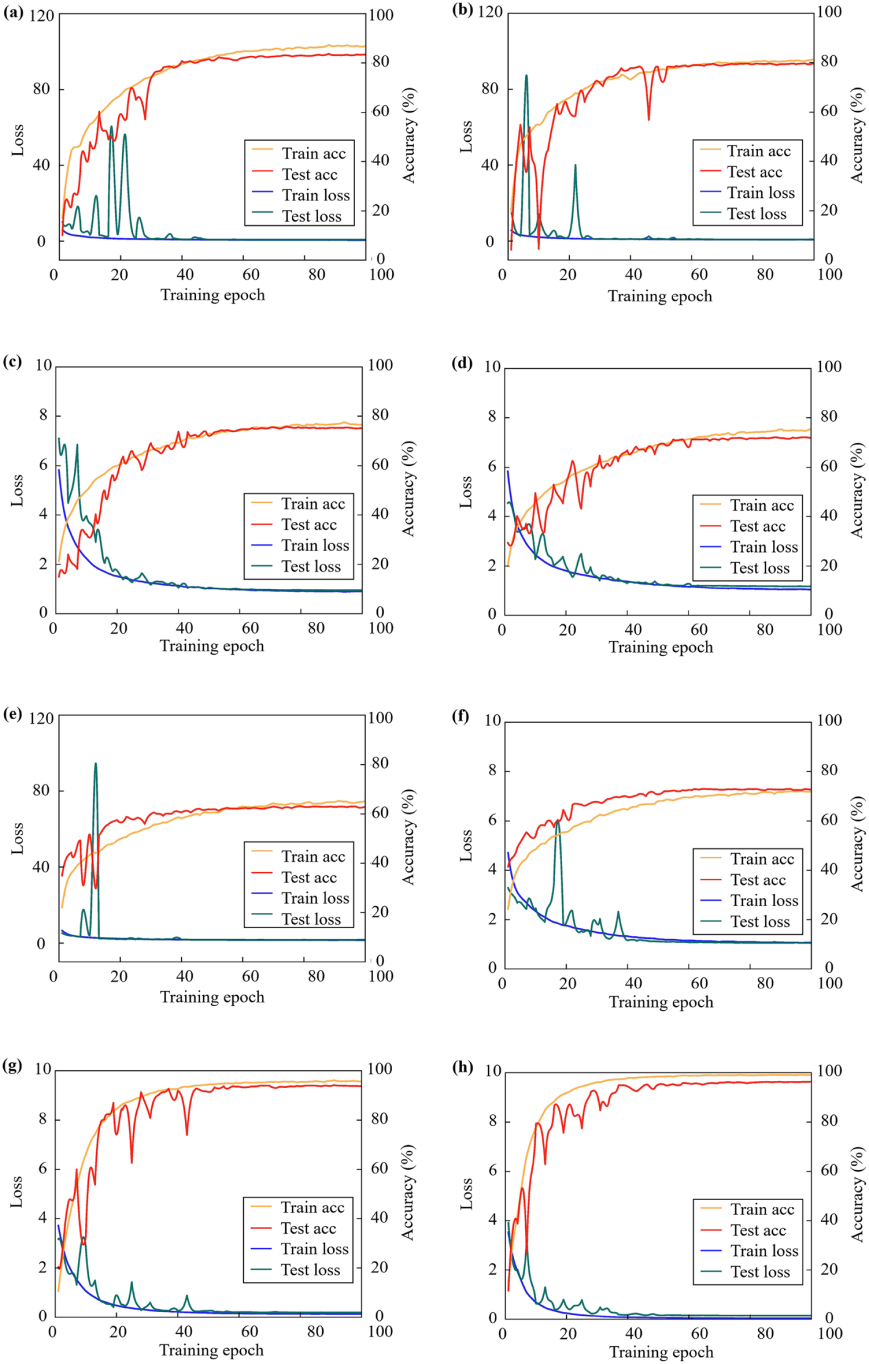
Loss and accuracy curves of eight pumpkin seed classification models in the training set and test set. **a** Vgg16, **b** Vgg19, **c** ResNet50, **d** ResNet101, **e** InceptionV3, **f** Xception, **g** 3DCNN, **h** PA-3DCNN

The classification results of 75 categories of pumpkin seeds by fine-tuning 6 pretrained models are shown in Table 4, and the loss and accuracy curves are shown in Fig. 8. The loss dropped rapidly as the model accuracy raised over 100 iterations. Comparing training and testing curves, six models revealed no negative transfer, overfitting or underfitting. The six models including Vgg16, Vgg19, ResNet50, ResNet101, InceptionV3, Xception achieved 83.42%, 80.66%, 75.89%, 70.83%, 61.38%, and 71.10% accuracies in the test set, respectively. Among them, the fine-tuned Vgg16 model attained the best classification effect. Since the structures of six transfer models were continuously adjusted to the optimal state according to the dataset, they had different depths. In this study, Vgg16 had the shallowest structure and fewer parameters. Generally, the features extracted by deep neural networks became richer due to more layers. In turn, the model classification accuracy would be higher [57]. However, the influence of factors such as dataset size, network depth, network structure and parameters could also lead to opposite conclusions [58, 59]. On the one hand, the above models were all two-dimensional convolutional neural networks, which paid more attention to extracting spatial features from spectral images. This made it difficult for the models to fully mine the features required for classification. On the other hand, the dataset in this study had a smaller scale and multiple categories. Meanwhile, a large amount of redundant information was contained in NIR spectral images. Therefore, the robustness of models was reduced by complex network structures and the accuracy was disturbed by noisy data. Simple models conversely performed better in classification of informative seed spectral images. Similarly, Wu et al. designed 1D deep neural networks including VGG-MODEL, RES-MODEL and INCEPTION-MODEL to fulfil effective classification of crop seeds based on NIR data. Among them, VGG-MODEL with the shallowest model depth worked best [46]. The authors also pointed out that the advantage of deep models was not in dealing with small datasets. Yang et al. employed a self-built CNN model to identify seed vitality [20]. And the model accuracy was better than ResNet18 with a deeper network structure, which verified that simple model structure can also handle information-rich spectral data. It can be seen that the optimal classification model structure is closely related to the size and distribution of the dataset.

**Table 4:** Results of pumpkin seed classification models based on spectral images

Model		Parameters (MB)	Training set accuracy (%)	Test set			
				Accuracy (%)	Precision (%)	Recall (%)	F1
Transfer pretrained models	Vgg16	254	86.84	83.42	83.58	83.42	83.34
	Vgg19	240	81.24	80.66	80.67	80.67	80.46
	ResNet50	849	76.67	75.89	75.86	75.89	75.58
	ResNet101	849	75.46	70.83	71.02	70.83	70.57
	InceptionV3	409	65.18	61.38	61.08	61.38	60.80
	Xception	1608	71.85	71.10	71.08	71.10	70.65
Ours	3DCNN	49	95.69	94.16	94.19	94.16	94.13
	PA-3DCNN	49	99.14	95.20	95.24	95.20	95.19

### Results of pumpkin seed classification model PA-3DCNN

The classification results of PA-3DCNN and 3DCNN without position attention module on 75 types of pumpkin seeds are shown in Table 4, and the loss and accuracy curves are shown in Fig. 8. The loss dropped rapidly within the first 30 iterations. After 60 iterations, the loss of two models approached 0 smoothly, which manifested good performance and stability. The test set accuracies of 3DCNN and PA-3DCNN achieved as high as 94.16% and 95.20%. In other words, the position attention module effectively ameliorated the classification performance by 1.05%. Not all regions in an image contributed equally to the classification task. The position attention module efficiently upgraded the classification accuracy by weighting features and selectively aggregating spatial features to find the most important parts in the network for processing. Zhu et al. added an attention mechanism module to the residual block of 3DResNet designed for high-dimensional hyperspectral images, which made better the model performance in effect [60]. It was confirmed that 3DCNN embedded with the attention mechanism had far-reaching prospects for the analysis and mining of HSI information.

Comparing the models constructed by machine learning methods and pretrained models, PA-3DCNN had the best classification effect. The classification accuracy of PA-3DCNN was respectively 1.98% and 11.78% higher than that of ELM and Vgg16, which effectually demonstrated its superiority in the classification of pumpkin seed varieties. Compared with 1DCNN and 2DCNN that only extract spectral or spatial features, 3DCNN utilized 3D convolution kernels to not only sample in the spatial domain but also along the spectral dimension, which simultaneously supplied a more efficient way to extract spatial and spectral information. In the meantime, the double convolution and pooling structure of PA-3DCNN made the number of parameters smaller and the model more compact. In addition, 3D hyperspectral data was utilized as input for direct end-to-end training, so that complex preprocessing and postprocessing were not required. This study showed that the combination of high-dimensional spectral images and 3DCNN had great perspective for the classification of multi-variety pumpkin seeds.

The performance of six classical deep learning networks in transfer learning was far worse compared to PA-3DCNN, which further proved that the complex network structure with numerous parameters was disadvantageous for recognizing pumpkin seeds. For traditional machine learning, ELM can make full use of the average spectrum, which was closest to the accuracy of PA-3DCNN. It strongly demonstrated that spectral information was more essential than spatial information in identifying seed categories. When the training data was sufficient and the model structure was properly designed, deep learning models usually received more satisfactory results. Therefore, it was crucial to select an appropriate model and algorithm according to dataset size and form of data.

### Results of PA-3DCNN transferability

**Fig. 9:**
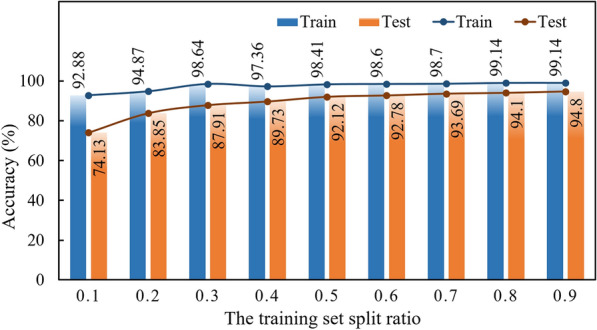
Accuracy of PA-3DCNN transfer model on the training set and test set under different division ratios of training set

In order to evaluate transferability and generalization ability of PA-3DCNN, the spectral images of 56 types of pumpkin seeds in 2021 were fed into the model in different proportions to build transfer models. The accuracy of training set and test set is shown in Fig. 9. When only 10% of samples were divided into the training set, the classification accuracy of PA-3DCNN transfer model was as high as 74.13%. As the training set gradually expanded, the accuracy of the transfer model was significantly promoted, which fully reflected that big data was the main driving force for the performance optimization of deep learning models. When 50% of spectral images were used as the training set, the classification accuracy of PA-3DCNN transfer model reached 92.12%. Although the accuracy had decreased compared to the original model, the accuracy of 92.12% was still acceptable considering the factors of training time and training cost. Therefore, transfer learning was a practicable method to improve the discriminative ability of multi-year crop seeds and reduce the cost of sample collection. In summary, PA-3DCNN was a classification model with strong generalization ability that can be used for multi-year pumpkin seeds.

### Visualization results

**Fig. 10:**
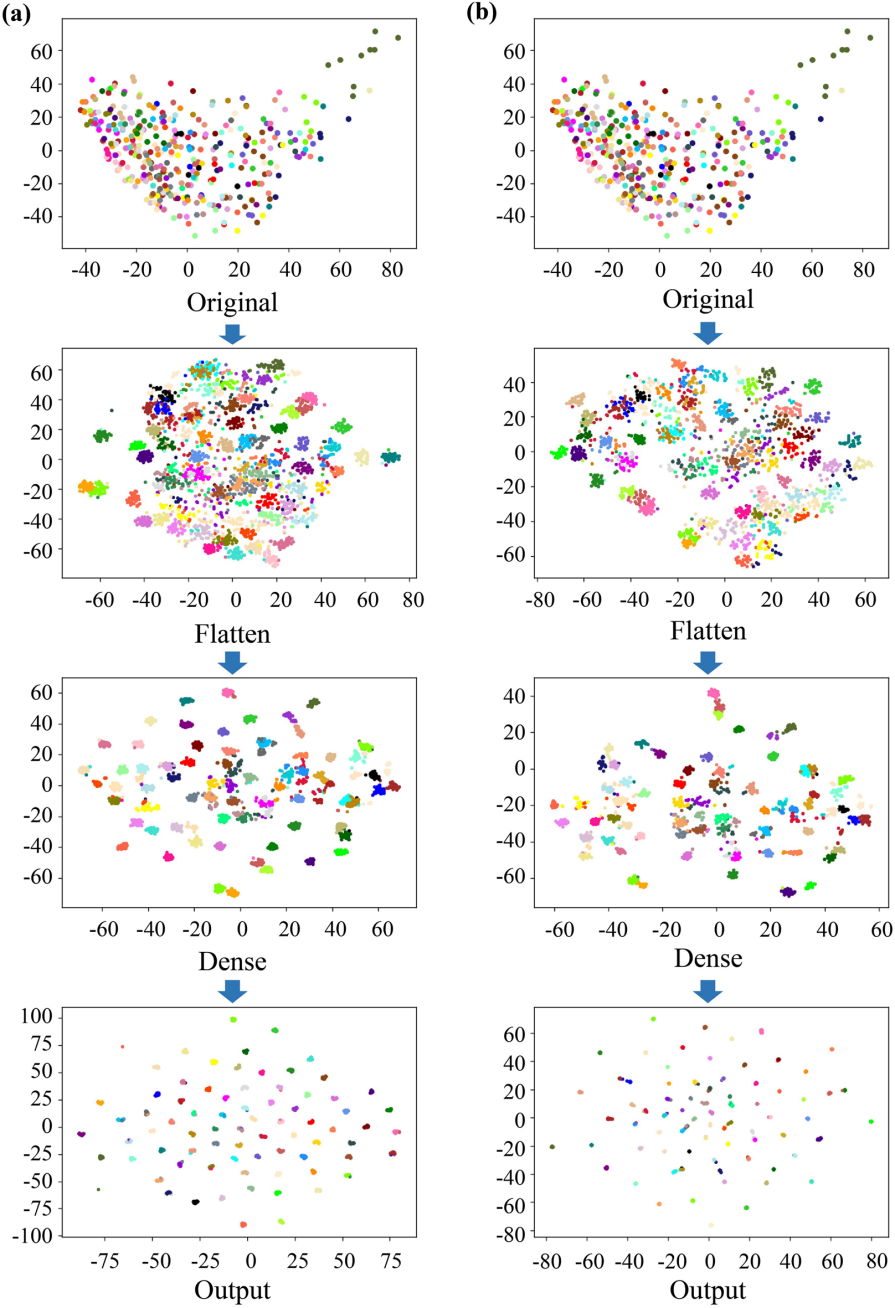
T-SNE visualization of the flattening layer, fully connected layer, input layer, and output layer of PA-3DCNN and 3DCNN. **a** T-SNE visualization of PA-3DCNN, **b** T-SNE visualization of 3DCNN

In order to demonstrate the effectiveness of deep learning and reveal the essence of spectral image classification, t-SNE visualization of the flattening layer, fully connected layer, input layer, and output layer of PA-3DCNN and 3DCNN are shown in Fig. 10. Different colors represented different types of pumpkin seeds. It can be seen from the figure that the spectral characteristic distribution of original pumpkin seeds was laborious to distinguish. After convolutional layers, the data points of pumpkin seeds gradually changed from overlapping to clearly separable, and each type can be clearly distinguished in the final output layer. On the other hand, since 3DCNN did not have position attention module, data points on the visualization map of its fully connected layer and output layer were more misclassified than PA-3DCNN, and data point coincidence was more serious. The t-SNE visualization map further indicated the good classification performance of PA-3DCNN.

**Fig. 11:**
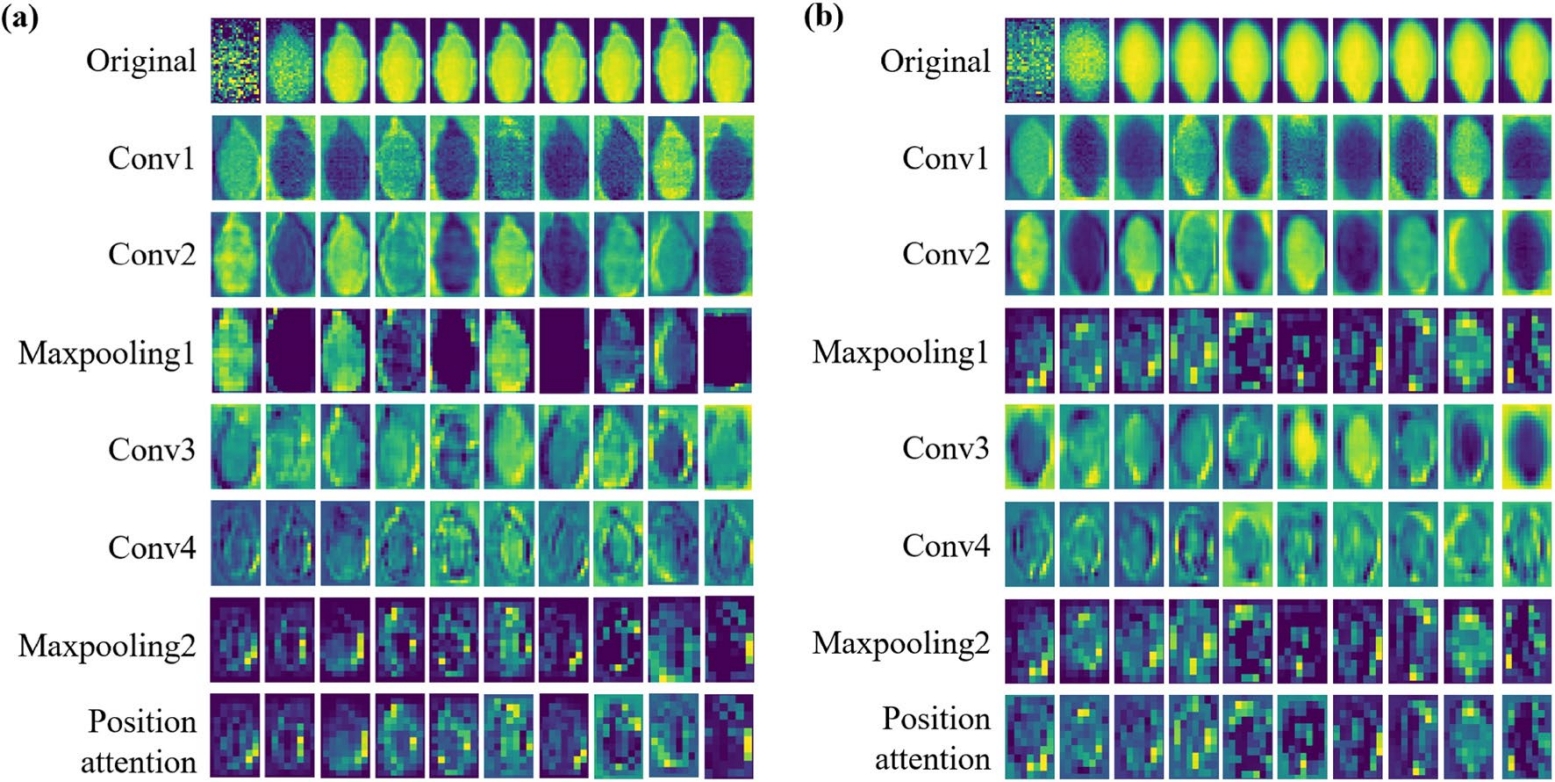
Feature visualization of the first 10 channels of convolutional layers and pooling layers of PA-3DCNN and PA-3DCNN transfer model. **a** Feature visualization of PA-3DCNN, **b** feature visualization of PA-3DCNN transfer model

The top 10 channel feature maps of convolutional and pooling layers of PA-3DCNN extracted and visualized by pseudo-color images are shown in Fig. 11a. There were differences in the images of dissimilar channels of original spectral images, which was different from RGB images. It proved that differing channels of high-dimensional spectral images offered a large amount of non-identical spectral information. The channel images from the first two convolutional layers recognized original seeds’ physical shape based on strengthening the edges of pumpkin seeds. As the depth of the network increased, the spectra of different positions of the whole seed were sequentially focused and locally analyzed in both spatial and spectral dimensions through convolution kernels. The feature maps of the convolutional and pooling layers gradually became abstract, but it was still noticeable that the edges of the pumpkin kernels were concentrated in the image as brighter pixels. Before features were flattened and transferred to the fully connected layer, the feature maps of the last layer had the approximate shape of pumpkin seeds as before, which revealed that the key and important spectral information was preserved.

Additionally, feature maps of the PA-3DCNN transfer model with a training set partition of 0.5 are shown in Fig. 11b. Since transfer learning employed the original model as a feature extractor, the feature extraction path of the transfer model was same as that of the corresponding self-built model. Comparing Fig. 11a and b, it was found that feature maps of different channels of each layer were basically similar, which further verified the feature extraction mode of transfer learning. All in all, the adoption of transfer learning to build models can not only ensure excellent classification results, but also help reduce computational pressure and training data requirements.

## Conclusion

In this study, Glycine, starch, fat, and soluble sugar were selected for the establishment of PLS regression model, which verified the strong correspondence between spectral characteristics and chemical components. Meanwhile, LDA was performed on the NIR average spectral data of 75 types of pumpkin seeds and good clustering results were obtained, which further confirmed the effectiveness of NIR spectral information for distinguishing seed varieties. After that, a pumpkin seed classification model PA-3DCNN was designed by fusing HSI technology and 3DCNN. The double convolution and pooling structure and position attention module were used to effectively boost its classification performance. The classification accuracy of 99.14% and 95.20% were respectively achieved on the training set and test set, which were 3.45% and 1.05% higher than that of 3DCNN. Compared with ELM and Vgg16, the classification accuracy was enhanced by 1.98% and 11.78% respectively, indicating that high-dimensional spectral images combined with 3DCNN had great potential in the classification of multi-variety pumpkin seeds.

Furthermore, the classification models based on traditional machine learning had received relatively good classification results, and the accuracy of ELM was the closest to that of PA-3DCNN. This evidenced that spectral information was more important than spatial information in identifying seed categories. On the contrary, the model constructed by transferring six classical deep learning networks had the worst classification performance, representing that the complex network structure with a large number of trainable parameters was unfavorable for recognizing NIR spectral images of pumpkin seeds. Additionally, in order to verify the generalization ability of PA-3DCNN, this study constructed a transfer model based on 56 types of pumpkin seeds in 2021, demonstrating that transfer learning was a feasible method to reduce the cost of sample collection and enhance the discrimination ability of multi-year crop seeds.

In conclusion, this study lent an efficient classification method for pumpkin seed varieties based on NIR spectral images. Meanwhile, the generalization performance of the model was verified by fine-tuning, which can be adopted for the classification of pumpkin seeds in multiple years. In the future, a more robust general model for the identification and classification of multi-year crop seeds should be constructed by building a spectral image database with various seeds and combining promising transfer learning.

## Data Availability

The original near-infrared hyperspectral images of pumpkins used and/or analyzed in the study can be obtained from the corresponding authors according to reasonable requirements. The code that supports the findings of this study is available from the corresponding author upon reasonable request.
